# Operative Therapie eines symptomatischen intraartikulären Osteoidosteoms (IAOO) in der Trochlea femoris

**DOI:** 10.1007/s00132-022-04218-9

**Published:** 2022-02-22

**Authors:** Daniel Schüll, Jörg Schmehl, Philipp Dalheimer

**Affiliations:** 1 Klinik für Orthopädie, Universität Tübingen, Tübingen, Deutschland; 2 Klinik für Radiologie, Universität Tübingen, Tübingen, Deutschland

**Keywords:** Knochentransplantation, Knorpel, Ödem, Neoplasien, Knochen, Radiofrequenzablation, Bone transplantation, Cartilage, Edema, Neoplasms, bone, Radiofrequency ablation

## Abstract

Intraartikuläre Osteoidosteome (IAOO) sind mit 10 % aller Osteoidosteome selten. Die atypischen klinischen und radiologischen Befunde führen meist zu einem langen Intervall zwischen Beschwerdebeginn und Diagnosestellung sowie Einleitung einer adäquaten Therapie. Dieser Fallbericht handelt von einer 32-jährigen Patientin, die seit Jahren intermittierende Kniegelenksschmerzen bei tiefer Beugung und selten nachts angibt. Bei IAOO in der Trochlea femoris war eine Radiofrequenzablation (RFA) aufgrund der direkt subchondralen Lage kontraindiziert. Daher erfolgte eine operative Sanierung mittels Knorpel-Knochen-Transplantation.

## Abstract (en)

The intra-articular osteoid osteoma (10% of cases) is a rare clinical finding. Based on atypical clinical signs and various radiological results the period to obtain a diagnosis and initiate the appropriate treatment is often very long. This case reports on a 32-year old female patient with knee pain on flexion and stress, which rarely occurred at night. Radiofrequency ablation (RFA) was contraindicated for IAOO in the trochlea femoris due to the direct subchondral location. Therefore, surgical rehabilitation by means of a cartilage-bone transplantation was carried out.

## Anamnese

Eine 32-jährige Patientin stellt sich mit vor 3 Jahren spontan begonnen Schmerzen am linksseitigen Kniegelenk erstmalig in unserer Klinik vor. Bis auf ein Anpralltrauma bei einem Handballspiel im Jahr 2016 werden weitere Verletzungen oder Erkrankungen das Kniegelenk betreffend verneint. Der Schmerzverlauf wird undulierend mit einem Mischbild aus Belastungsschmerzen und Ruheschmerzen beschrieben. Bei Beschwerdebeginn bestanden zusätzlich nächtliche Beschwerden. Anlaufbeschwerden oder eine Morgensteifigkeit werden verneint. Über den Beschwerdezeitraum hinweg seien die Beschwerden insgesamt progredient und die auftretenden Schmerzepisoden gehäuft. Gleichzeitig zeigen sich kürzere und seltenere beschwerdefreie Episoden. Das Punctum maximum des als stechend beschriebenen Schmerzes wird im Bereich der Patella angegeben. Über die spontan auftretenden Beschwerden hinaus kann durch Flexionsbewegungen über 90° hinaus eine Schmerzprovokation im Bereich der Patella generiert werden. Eine exakte Lokalisation kann nicht angegeben werden.

## Befunde

### Klinisch

Bei der klinischen Untersuchung zeigt sich ein flüssiges Gangbild. Haut und Weichteile reizlos, keine Schwellung Rötung oder Überwärmung. Diskreter Gelenkerguss. Kein Druckschmerz über den Gelenkspalt. Diskreter Patellaverschiebeschmerz mit positivem Zohlen-Zeichen. Extension/Flexion 0‑0-90°, endgradig schmerzhaft. Bandapparat stabil. Meniskuszeichen negativ.

### Radiologisch

**Figure 1:**
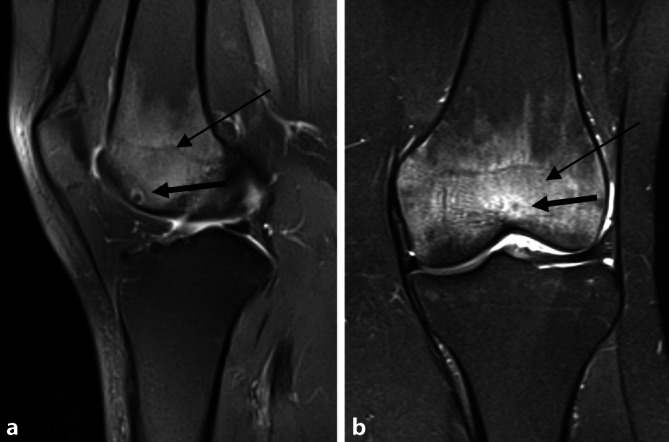

MRT des Kniegelenks zum Zeitpunkt der Operation sowie 3 Jahre zuvor. Zu allen Zeitpunkten zeigt sich ein hyperintenses Ödem in der T2-Wichtung im distalen Femur bei Nachweis eines Nidus in der Trochlea femoris (Abb. 1 und 3). Der Nidus ist in der T2-Wichtung charakterisiert durch eine Hyperintensität mit meist umgebener signalarmer Sklerosezone (Abb. 3). Im vorliegenden Fall weist der Nidus einen Bezug zur subchondralen Knochenlamelle und somit auch eine unmittelbare Nähe zum Gelenkknorpel auf (Abb. 3). Ein Gelenkerguss besteht nicht.

## Diagnose

**Figure 2:**
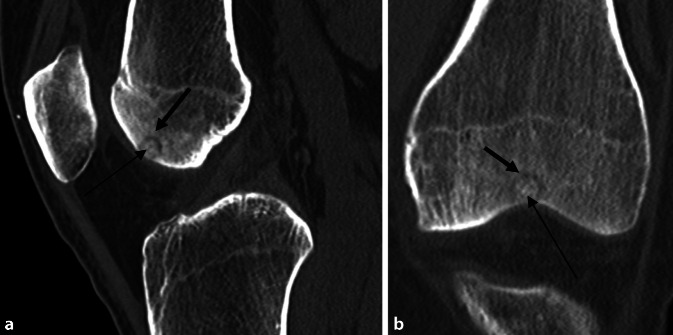

Die CT-Diagnostik bestätigte den Befund einer subchondralen, epiphysären Läsion interkondylär, vereinbar mit einem Osteoidosteom bei hyodensem Nidus mit hyperdenser Kalzifizierung (Abb. 2). Das initial durchgeführte Röntgenbild (Abb. 4) zeigt indes keine wegweisenden Auffälligkeiten.

**Figure 3:**
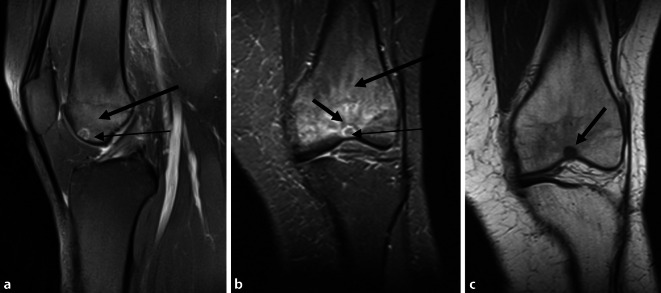

**Figure 4:**
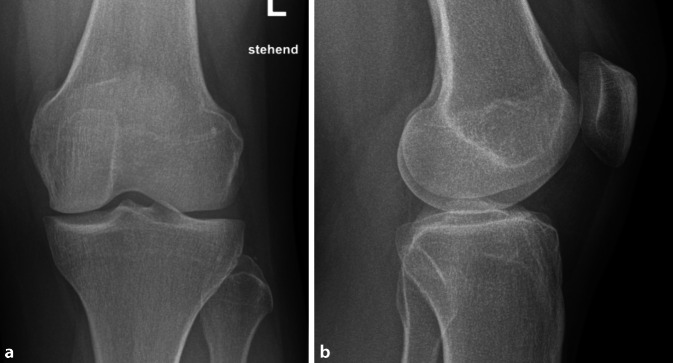

## Therapie und Verlauf

Von Beschwerdebeginn bis zur Erstvorstellung in unserer Klinik erfolgte auf Grundlage des unklaren Knochenmarködems und subchondralen Defekts in der MRT eine Therapie mit Calcium und Vitamin D sowie Bisphosphonaten durch einen niedergelassenen Kollegen. Weiterhin wurde durch den behandelnden Orthopäden die Gabe von Acetylsalicylsäure (ASS) initiiert. Hier ergab sich jedoch keine merkliche Verbesserung der Beschwerden. Im weiteren Verlauf erfolgte schließlich die symptomatische Therapie mit verschiedenen Nichtsteroidale Antirheumatika (NSAR) bei begleitend steigendem Beschwerdeniveau.

**Figure 5:**
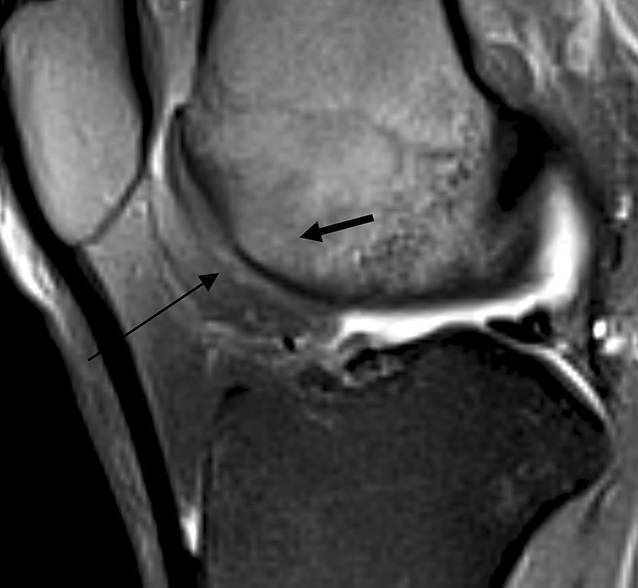

Bei progredienten MRT-Befunden und der Diagnosesicherung des Osteoidosteoms mittels CT (Abb. 2) wurde die Radiofrequenzablation mit den Kollegen der Radiologie ausführlich diskutiert. Diese war aber aufgrund der Nähe des Nidus zur subchondralen Lamelle und MRT-morphologisch vermutetem Kontakt zum Gelenkknorpel kontraindiziert (Abb. 5).

Wir stellten daher die Indikation zur Arthroskopie des Kniegelenkes mit Resektion der unklaren Raumforderung und Transplantation eines Knorpel-Knochen-Zylinders. Es wurde präoperativ mit der Patientin besprochen, dass das Aufsuchen der Defektzone über eine Reaktion des Gelenkknorpels möglich sein sollte, andernfalls würde anhand des makroskopischen Bildes sowie einer intraoperativen Röntgenaufnahme der Nidus lokalisiert werden.

**Figure 6:**
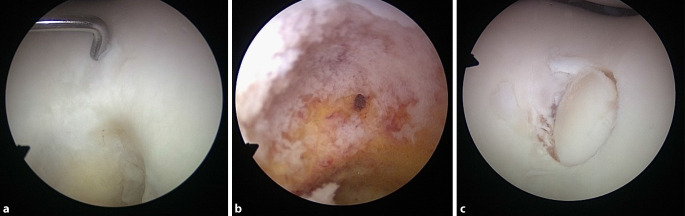

Arthroskopisch zeigte sich intraoperativ bei einer Ansicht des Gelenkknorpels lediglich eine kleine zentrale Auffälligkeit in der Trochlea femoris. Bei näherer Prüfung des Gelenkknorpels mittels Tasthaken konnte hier eine deutliche Aufweichung festgestellt werden. Die Randzone war mittels Tasthaken dabei abgrenzbar (Abb. 6a). Es wurde ein Knorpel-Knochen-Zylinder mit dem Durchmesser von 8 mm im Bereich des Defektes entnommen. Der subchondrale Knochen zeigte Veränderungen der Spongiosa (Abb. 6b). Diese wurden vollständig reseziert und zur histopathologischen Untersuchung eingeschickt. Zur Defektauffüllung wurde letztendlich ein Knorpel-Knochen-Zylinder aus dem proximalen Bereich der medialen Femurkondyle entnommen und in die Defektzone, leicht unter das Niveau des übrigen Gelenkknorpels, eingesetzt (Abb. 6c).

Die histopathologische Aufarbeitung der Knorpel-Knochen-Stanze aus der Trochlea femoris ergab ein 5 mm messendes Osteoidosteom. Eine weitere Behandlung war nach vollständiger Entfernung nicht indiziert.

Es erfolgte eine 6‑wöchige Teilbelastung der Extremität mit 20 kg Körpergewicht mit anschließender Aufbelastung. Begleitend wurde zur Vermeidung einer Spitzenbelastung im patellofemoralen Gleitlager eine 4‑Punkt-Rahmenorthese mit Flexionslimitierung von 90° verordnet. Bei der ausgeprägt sportlichen Patientin mit hohem Bewegungsbedarf erfüllte die Rahmenorthese auch den Zweck einer allgemeinen postoperativen Aktivitätsreduktion. Nach 12 Wochen war die Vollbelastung wieder erreicht.

Bei der Halbjahreskontrolle zeigte sich in der klinischen Untersuchung ein reizloses Kniegelenk ohne Schwellung mit jedoch noch diskretem Erguss. Die Beweglichkeit war sowohl passiv als auch aktiv im Seitenvergleich frei. Ein leichter Schmerz konnte lediglich bei der Patellaverschiebung sowie dem Zohlen-Zeichen ausgelöst werden. Zudem gibt die Patientin leicht Beschwerden in der tiefen Hocke an. Gelegentlich verspüre sie ein Knacken in tiefer Beugung. Die Patientin ist sowohl privat als auch beruflich nicht eingeschränkt. Die nächtlichen Schmerzen seien nicht mehr aufgetreten. Sportliche Belastungen (wie z. B. Radfahren) waren wieder möglich.

**Figure 7:**
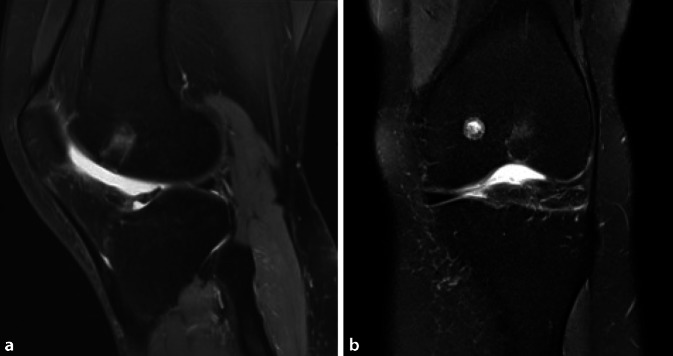

Die MRT-Verlaufskontrolle (Abb. 7) zeigte einen vollständig eingeheilten Knorpel-Knochen-Zylinder. Das in den Voraufnahmen sichtbare Knochenödem zeigte sich vollständig regredient (Abb. 3).

## Diskussion

Das Osteoidosteom ist mit einem Anteil von 10 % aller benigner Knochentumoren ein vergleichsweise häufiger Knochentumor, mit gehäuftem Auftreten vom 10.–25. Lebensjahr [2]. Charakteristisch bestehen Nachtschmerzen und Ruheschmerzen und ein Ansprechen auf Salicylate. 10–20 % aller benignen Knochentumoren sind Osteoidosteome mit überwiegender Lokalisation an Dia- und Metaphysen langer Röhrenknochen. Intraartikuläre Osteoidosteome (IAOO) finden sich mit etwa 10 % aller Osteoidosteome nur selten [1]. Aufgrund der untypischen Lage und einhergehendem atypischem Beschwerdebild stellen diese eine größere diagnostische Herausforderung dar und führen häufig zu einem langen Verlauf bis zur Diagnosestellung. Im vorliegenden Fall lag das Intervall bei >3 Jahre nach Beschwerdeerstmanifestation. In der Literatur finden sich Intervalle zwischen 4 Monaten und 5 Jahren bis zur Diagnosestellung [3].

Diagnostische Schwierigkeit bereitet dabei ein Mischbild aus tumorbedingten Beschwerden, verursacht durch eine nächtliche Mehrproduktion an Prostaglandinen im Nidus [4], sowie diffusen Gelenkbeschwerden. Hierbei treten die Gelenkbeschwerden meist in den Vordergrund und können aufgrund ihrer Morphologie Monarthritis, Monarthrose, Synovialitis oder anderen Grunderkrankungen ähneln und so die eigentliche Diagnose weiter verschleiern [1, 5]. Klassische radiologische Hinweise, wie ein vorhandener Nidus, sind in 50–70 % der Fälle sehr variabel ausgeprägt oder nicht zu finden [3]. Klassische Röntgenbilder ergeben oftmals keine wegweisenden Befunde. In der MRT-Diagnostik ergibt sich häufig ein variables Knochenödem [6]. Beim Erkennen eines Nidus zeigen sich meist variable Erscheinungsmuster je nach Läsionsalter, Sklerosierung, Durchblutung und Größe [2].

Ausgehend von diesen Schwierigkeiten bedarf die Diagnosestellung einer sorgfältigen Strategie und Aufarbeitung, um eine unnötige Verzögerung der Diagnosesicherung zu vermeiden. In unserem Fall erbrachte letztlich die kombinierte Bildgebung aus MRT, CT und klassischer Röntgenbildgebung, mit hypodensem Nidus innerhalb einer hyperdensen Sklerosewolke in der CT und einer entsprechenden nidalen Kontrastmittelaufnahme mit perifokalem Ödem in der MRT, in Verbindung mit der atypischen klinischen Symptomatik aus Nachtschmerzen und bewegungsabhängigen Beschwerden die Verdachtsdiagnose eines intraartikulären Osteoidosteoms.

Hinsichtlich der therapeutischen Optionen stellt die Radiofrequenzablation (RFA) als minimal-invasives Verfahren und Erfolgsraten von 90 % bei entsprechenden Lokalisationen das Mittel der Wahl dar [4, 7]. Aufgrund der intraartikulären und subchondralen Lage war dieses Verfahren, bei möglichen Knorpelschäden durch die Hitzeeinwirkung, kontraindiziert [8].

Daher stellten wir bei dem vorliegenden Befund die Indikation zur Arthroskopie mit vollständiger Resektion des Nidus und anschließender Knorpelersatztherapie im Bereich der Trochlea femoris. Zu den wichtigsten operativen Optionen der Knorpelersatztherapien zählen die Mikrofrakturierung, die osteochondrale autologe Knorpel-Knochen-Transplantation, die Transplantation osteochondraler Allografts, die autologe Chondrozytentransplantation sowie die autologe matrixinduzierte Chondrogenese [9]. Bei radiologisch nachgewiesenem subchondralem Knochendefekt besitzt die Mikrofrakturierung in diesem Fall keinen Stellenwert. Aufgrund der aktuellen Studienlage sowie der zu erwartenden Defektgröße wurde das Verfahren der osteochondralen autologen Knorpel-Knochen-Transplantation gewählt [2, 10]. Die präoperative Planung zeigte bereits, dass bei der vorliegenden Defektgröße eine Knochenstanze mit dem Durchmesser von 8–10 mm zur vollständigen Sanierung ausreicht. Somit war der komplette Befund mit einer Knorpel-Knochen-Stanze zu kurieren.

## Fazit für die Praxis

- Intraartikuläre Osteoidosteome (IAOO) sind seltene Befunde.
- Bei diffuser Beschwerdesymptomatik mit einem Mischbild aus bewegungsabhängigen Beschwerden und Nachtschmerzen sollte das IAOO als Differenzialdiagnose in Betracht gezogen werden.
- Zur Vermeidung langer Diagnoseintervalle sollten zielgerichtet eine MRT- und CT-Bildgebung erfolgen.
- Je nach Befund muss eine individuelle Therapieplanung mit dem Patienten erfolgen.
- Bei direkt subchondral gelegenen Defekten stellt die Resektion der unklaren Raumforderung und Transplantation eines Knorpel-Knochen-Zylinders eine suffiziente Behandlungsmethode dar.

## Abkürzungen

ASS: Acetylsalicylsäure
IAOO: Intraartikuläres Osteoidosteom
NSAR: Nichtsteroidale Antirheumatika
RFA: Radiofrequenzablation
